# Rapid Determination of Colistin Susceptibility by Flow Cytometry Directly from Positive Urine Samples—Preliminary Results

**DOI:** 10.3390/ijms26030883

**Published:** 2025-01-21

**Authors:** Daniela Fonseca-Silva, Rosário Gomes, Inês Martins-Oliveira, Ana Silva-Dias, Maria Helena Ramos, Cidália Pina-Vaz

**Affiliations:** 1 Department of Clinical Pathology, Instituto Português de Oncologia, 4200-072 Porto, Portugal; daniela.sfsilva@gmail.com; 2 FASTinov SA, 4200-135 Porto, Portugal; rosariogomes@fastinov.com (R.G.); ioliveira@fastinov.com (I.M.-O.); anadias@fastinov.com (A.S.-D.); 3 RISE—Health Department of Pathology, Faculty of Medicine, University of Porto, 4200-319 Porto, Portugal; 4 Department of Microbiology, Centro Hospitalar e Universitário do Porto, 4099-001 Porto, Portugal; maria.helena.s.ramos@gmail.com; 5 Department of Microbiology, Faculty of Medicine, University of Porto, 4200-319 Porto, Portugal

**Keywords:** colistin, rapid antimicrobial susceptibility test, flow cytometry, antimicrobial resistance, urinary tract infections

## Abstract

Urinary tract infections caused by Gram-negative bacteria (GNB) are among the most common infections and a significant cause of sepsis. The increasing prevalence of multidrug-resistant (MDR) bacteria poses challenges to empirical treatment. Colistin may be used a last-resort antibiotic for treating MDR infections, but this requires the rapid determination of susceptibility to colistin. Traditional susceptibility testing methods can take up to 48 h, and there are specific challenges in determining colistin susceptibility. This study evaluates a novel, rapid method for determining colistin susceptibility directly from positive urine samples using the FASTcolistin MIC kit from FASTinov^®^. A total of 100 urine samples positive for Gram-negative bacilli when screened by the UF-1000i system were included in this study. After a simple sample prep, the same bacterial suspension was used for identification on MALDI-TOF and inoculated in the FASTcolistin MIC panel for our AST; after incubation at 37 °C for 1 h, it was analyzed via flow cytometry using a CytoFLEX cytometer (Beckman Coulter, Brea, CA, USA). The categorical susceptibility to colistin according to EUCAST or CLSI standards as well as the MIC values were given by bioFAST software (bioFAST 2.0). The essential agreement (EA) and bias were calculated. Different species of Enterobacterales, *Pseudomonas aeruginosa*, and *Acinetobacter* spp. were correctly identified by MALDI-TOF directly from the FASTcolistin MIC sample prep. The essential agreement between the two methods was 99%, with a bias of −17%. Both identification and susceptibility were obtained in less than 2 h. This study presents a rapid and accurate method for determining colistin MIC directly from urine samples. The shortness of time required to produce a result, 2 h versus 48 h with the conventional methods, will significantly impact treatment decisions, especially in urinary tract infections difficult to treat.

## 1. Introduction

Urinary tract infections (UTIs) are the second most common type of infection worldwide, affecting 150 million people each year, with significant morbidity and high medical costs (e.g., it has been estimated that the economic burden of recurrent UTIs in the United States is more than USD 5 billion each year) [1], and with a higher incidence in females. Gram-negative aerobic bacteria (GNB), particularly *Escherichia coli* (responsible for 75–90% of UTIs), are the predominant causative agent, followed by *Klebsiella pneumoniae*, Proteus, and *Pseudomonas* spp. [2].

Up to 50–70% of women experience a UTI within their lifetime, and of these, 40% will have a recurrence [3]. The use of urinary catheters and underlying conditions such as diabetes further increase the risk of recurrence. UTIs are a common cause of sepsis, accounting for 19.8% of cases [3]. Antimicrobial resistance (AMR) in UTIs pathogens is a growing concern, exacerbating the challenge of treating both primary and recurrent infections. The prevalence of carbapenem-resistant *Enterobacteriaceae* (CRE) is increasing, threatening the efficacy of carbapenem antibiotics, commonly considered the last line of defense against multidrug-resistant strains (MDRs) of Gram-negative bacteria [4]. This rise in AMR is linked to the widespread and often inappropriate use of antibiotics in both medical and agricultural sectors [5]. The growing prevalence of AMR leads to longer treatment times, higher healthcare costs, and increased morbidity, especially in recurrent infections where treatment options become limited [6]. The need for alternative strategies is leading people to look back to old therapies like colistin.

Colistin (polymyxin E) is used as a last resort for the treatment of critically ill patients infected with multidrug-resistant (MDR) GNB infections [7]. These patients are usually extremely ill and require a rapid AST result. Colistin can be used intravenously, inhaled, used intrathecally, or even given through intravesical irrigation [8]. Determining colistin susceptibility is considered a challenge in traditional methods as it adheres to plastic (automated methods, disks, and the E-test are not recommended by EUCAST or CLSI guidelines), with broth microdilution being the only gold standard for determining the colistin MIC (minimum inhibitory concentration), although laborious and time consuming [9].

Rapid antimicrobial susceptibility tests (ASTs) for colistin using FASTinov technology from isolated colonies [10] and directly from positive blood cultures [11] presented excellent results and saved 1–2 days, respectively. FASTinov technology is a disruptive technology, which uses flow cytometry as a basis, not dependent on bacterial growth, in contrast to most ASTs on the market. It is based on the detection of bacteria cell lesions after a short incubation time with the drugs [12]. It has been used especially in critical situations like sepsis, but here we describe its first application directly in positive urine samples.

UF-1000i is a urine flow cytometer from Sysmex Corporation (Kobe, Japan) (Sysmex^®^), which uses a diode laser to quantify sediment in two analytic channels and a fluorescent dye, which stains DNA. One channel analyzes only the microbial contents of the urine, while the other analyzes RBCs, WBCs, casts, and other non-microbial sediment. The staining agent is a fluorescent polymethine dye that binds to DNA. After staining, the particles are transported to a flow cell and are irradiated by a semiconducting laser (λ 635 nm) [13]. Its use to screen urine and select infected samples showed a sensitivity/specificity of 81.8/91.1% in detecting Gram-negative bacteria [14], allowing for the first example a rapid AST, where culturing was no longer performed.

A rapid AST requires the rapid identification of microorganisms (ID). MALDI-TOF technology, with its rapid ability to ID, allows for the identification of microorganisms from colonies. Excellent ID results were obtained when using the sample prep described on FASTinov kits directly from positive blood cultures, avoiding sub-culturing [15].

Preliminary data obtained using clinical samples from patients with UTIs and urine samples inoculated with well-known characterized bacteria, screened positive for GNB using the Sysmex 1000i system, are presented in this article regarding the direct ID of bacteria and the determination of the susceptibility to colistin via the FASTcolistin MIC in less than 2 h.

## 2. Results

MALDI-TOF correctly identified 100% of the samples in at least one of the spots. Table 1 shows the distributions of species in clinical and inoculated samples and the intervals of MIC values obtained by microdilution in the studied strains.

**Figure 1:**
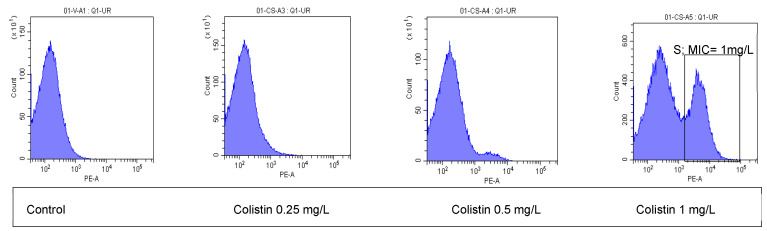
Flow cytometry histograms of an example of a susceptible (S) strain after exposure to different concentrations of colistin ranging from 0.25 mg/L to 1 mg/L; the control is the histogram of cells not exposed to the drug; all the cells represented were exposed to the fluorescent probe. After 1 mg/L of colistin, the histogram shows a drift of the population to the right, meaning that cell membrane lesions were forming as colistin was being effective (MIC value).

Flow cytometry analysis of each strain was carried out in 20 min instrument time and the result was immediately obtained using the proprietary bioFAST software. Categorization of the phenotype and MIC value was performed for each strain and compared with microdilution. An example of cytometry histograms of a susceptible strain is presented in Figure 1. In the case of a resistant phenotype, the histogram is similar to that of the control, with at least colistin concentration of 4 mg/L, (over the 2 mg/L breakpoint concentration of both the EUCAST and CLSI protocols) [16,17]. Ten strains were resistant (MIC > 2 mg/L) and all correctly detected by the FASTcolistin MIC kit.

The essential agreement between the FASTcolistin MIC and reference methods was 99%, with a bias of −17% (Table 2).

## 3. Discussion

UTIs, primarily caused by Gram-negative bacteria, pose a significant public health challenge due to their prevalence and risk of life-threatening sepsis. Patients with recurrent UTIs are submitted to several antimicrobial treatments per year, meaning they often develop multidrug-resistant strains with no good therapeutic options. Rapid and accurate diagnostic techniques are essential to guide targeted therapy, reducing the reliance on broad-spectrum antibiotics, which contribute to antimicrobial resistance.

A standard AST from urine is time-consuming, as it requires first sub-culturing for 24 h before identification and the AST can be performed. Matrix-assisted laser desorption ionization–time of flight mass spectrometry (MALDI-TOF) performed from colonies provides an immediate and accurate ID one day before an AST. It typically takes 2 days to complete a microbiology report, often resulting in limited and delayed clinical benefit.

Syxmex developed a method for the screening analysis of urine that removes the need to smear negative samples, reducing the technical work required, as they are the majority. Clinical samples used in this study, screened positive with UF-1000i for GNB > 10^5^/mL, were used to ID and AST directly from urine after the FASTinov sample prep.

The FASTcolistin MIC presented in this study offers a rapid, accurate method that could serve as an alternative to traditional AST methods, with a significant reduction in the time-to-result to under 2 h. This approach could substantially impact UTI management, reducing the time-to-result and time to therapy, which will be reflected in patient outcomes. Patients put forward for colistin treatment are always critical patients without a lot of therapeutic options. The rapid detection of resistance will avoid therapeutic failure with a high risk of mortality.

Among the limitations of this study are the possibility that urine samples may be polymicrobial. Despite the Sysmex system and MALDI TOF detecting more than one population, polymicrobial samples may challenge them when we work directly with clinical samples. The other concern is the need for the method to be performed in a lab and with a few manual steps, although those are simple and could be performed for any lab technician.

For now, the Fastinov Colistin kit is only optimized for colistin E; in the future, it should be extended to other drugs and polymyxins, such as colistin B, widely used in the USA.

Recently, methodologies for rapid ASTs in urine, like the PA-100 AST System, have been described. They include the susceptibility to only five commonly prescribed antibiotics for the treatment of uncomplicated UTIs: amoxicillin/clavulanic acid, ciprofloxacin, fosfomycin, nitrofurantoin, and trimethoprim. However, they can be performed easily in outpatient settings, while FASTinov technology, at the moment, can only be used in a clinical lab.

Further multicenter validation studies with FASTcolistin MIC testing in clinical urine samples are ongoing, as well as the expansion of the application of this technology to other antimicrobials (beta-lactamics, quinolones, aminoglycosides, fosfomycin, and nitrofurantoin), similar to the process followed with positive blood cultures [18].

In this work, financial aspects were not considered as this kit is not yet on the market [19].

## 4. Material and Methods

### 4.1. Urine Samples

**Figure 2:**
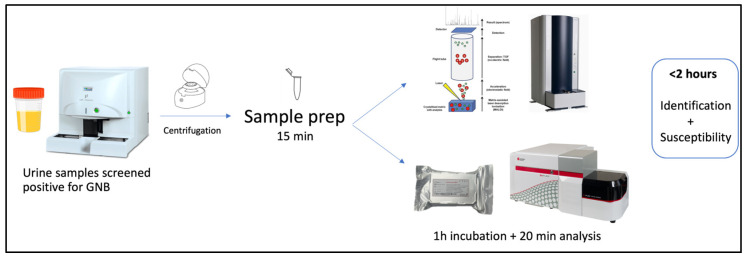
Workflow of this study performed with urine samples.

Patients were recruited from the Urology Department of a tertiary center, after being admitted with UTIs. Ninety urine samples collected in sterile VACUETTE^®^ tubes were analyzed on the UF-1000i system (Syxmex, Kobe, Japan), and thirty-nine positive for GNB (>10^5^/mL) were included in this study. Samples with Gram-positive microorganisms/contamination or with <10^5^/mL were excluded. Sixty-one urine samples collected from healthy volunteers were filtered with a 22 µm filter (Q-Max^®^, Frilabo, Maia, Portugal) and each one inoculated with characterized GNB strains from the cryobanked collection of the Microbiology Department, Porto Medical School (Portugal) before being incubated overnight. Two ATCC strains were included: *E. coli* 8739 with an MIC to colistin of 0.5 mg/L, and *E. coli* 13846, positive for mcr1 with an MIC to colistin >64 mg/L. The workflow is presented in Figure 2.

### 4.2. Sample Preparation and Identification

After centrifugation, 10 mL of urine sample was centrifuged at 3000 rpm for 10 min, supernatant was rejected, and the pellet was resuspended in 1 mL of sterile saline solution. Then, 50 µL of hemolytic agent (tergitol 10%) was added and vortexed for 10 s. The tube was centrifuged at 13,000 rpm for 1 min; the supernatant was thew rejected and the pellet resuspended in 500 µL of saline solution. This suspension was carefully poured on top of 500 µL of Histopaque^®^ (Sigma-Aldrich, St. Louis, MO, USA) (a density gradient medium) and centrifuged again at the same speed and for the same time. This achieved the removal of the remaining urine cells and debris, preserving bacteria. Two spots of pellets were inoculated in the Bruker MALDI Biotyper CA System (Bruker Daltonics, Billerica, MA, USA), and we performed an ID in a similar way to that for a colony.

### 4.3. Incubation with Colistin and Fluorescent Probe

A bacterial pellet was resuspended in 500 µL of sterile saline solution, adjusted to 0.5 McFarland suspension, and diluted (1 mL in 7 mL) with Muller–Hinton cation-adjusted broth medium (Lioflchem, Ref.26177, Roseto degli Abruzzi, TE, Italy). The FASTcolistin MIC panel (96-well panel) from FASTinov^®^ containing 9 concentrations of colistin dilutions, from the 3rd to the 11th wells, halved each time (0.25–64 mg/L), and a fluorescent probe (patent protected) was inoculated. Three controls were included: autofluorescence—1st well without drug or fluorescence probe; viability control—2nd well without drug but with fluorescence probe; and a dead control—12th well with benzydamine and the fluorescence probe, in order to be sure that the probe is active. Incubation was performed for 1 h at 35 ± 2 °C with shaking (550 rpm/min).

### 4.4. Flow Cytometry Analysis

Flow cytometry was performed using a CytoFLEX cytometer equipped with one blue laser (488 nm; output, 50 mW; beam spot size, 5 by 80 μm). The instrument has three fluorescence channels: 525/40 BP, 585/42 BP, and 690/50 BP. It is also equipped with a plate reader for the automatic analysis of each panel; each sample takes 15 min instrument time. Flow cytometry is similar to a fluorescence microscope but can analyze a significant number of cells in a few seconds, recording their size, complexity, and fluorescence intensity. Using optimized fluorochromes, we are able to see the damage that is being caused by drugs to bacteria after a short incubation time. As the bacteria are being “killed”, the population seen in the cytometry forward scatter line is moved to the right. This is noticeable in histograms. Although this change in cells is quite visual, Fastinov has software with its own algorithm, which interprets the changes and compares them to a large database, interpreting the results as susceptibility or resistance and guiding the report it produces.

The flow cytometer was used in the slow mode, and data analysis was automated using bioFAST software, a FASTinov proprietary software, which provided the MIC value and susceptibility category based on EUCAST and CLSI standards. A multiparametric analysis compared the treated cells with the non-treated cells (control of viability) regarding the sizes and complexity of the cells and the intensity of fluorescence, especially at 690/50 BP (PE-E).

### 4.5. Reference Method for Colistin Susceptibility

In parallel, all urine samples analyzed were sub-cultured, colonies identified using MALDI-TOF technology, and colistin susceptibility tested using the standard broth microdilution method, with the results interpreted according to the EUCAST/CLSI guidelines [16,19]. Briefly, a serial two-fold dilution of colistin was prepared in Muller–Hinton broth (MHB), and 100 µL of each colistin dilution was dispensed to the corresponding wells in a 96-well plate, with concentrations ranging from 0.25 mg/L to 64 mg/L. A standardized bacterial suspension was prepared by adjusting the turbidity of the inoculum to 0.5 McFarland standard (approximately 1–2 × 10⁸ CFU/mL) and diluting the suspension in MHB to achieve a final inoculum concentration of 5 × 10⁵ CFU/mL in each well. The bacterial inoculum was added to each well (100 µL) containing colistin, bringing the final volume in each well to 200 µL. The microplate was incubated for 24 h at 35 ± 2 °C, and we visually check each well for bacterial growth by observing turbidity. The MIC was defined as the lowest concentration of colistin that completely inhibited visible bacterial growth.

### 4.6. Statistical Analysis

The essential agreement (EA) and bias of the FASTcolistin MIC were calculated according to International Organization for Standardization [20] taking microdilution as the standard.

## 5. Conclusions

The high frequency of recurrent UTIs, combined with the increasing prevalence of antimicrobial resistance, poses a substantial public health challenge. Urgent measures are needed to promote antibiotic stewardship and enhance diagnostic tools, which could help with patient treatment and prevent AMR. The FASTcolistin MIC kit is the fastest in the world when seeking to accurately determine the MIC directly from a positive urine sample.

## Figures and Tables

**Table 1:** Distribution of bacterial species analyzed on the FASTcolistin MIC, including patient samples and inoculated samples in urine; interval of minimal inhibitory concentrations (MIC—mg/L) obtained per species by microdilution.

	Species	Patients	Inoculated	Total	MIC
*Enterobacterales*	*Escherichia coli*	23	7	30	0.125->64
	*Klebsiella pneumoniae*	13	6	19	0.5–2
	*Klebsiella aerogenes*	2	1	3	1–2
	*Serratia marcescens*		1	1	8
	*Proteus mirabilis*	1	2	3	>64
	*Providencia rettgeri*		1	1	4
	Total	39	18	57	
No fermenters	*Pseudomonas aeruginosa*		30	30	0.25–4
	*Acinetobacter baumannii*		12	12	1–4
	*Enterobacter cloacae*		1	1	2
	Total	0	43	43	
Total studied		39	61	100	

**Table 2:** Correlation between the determination of the minimum inhibitory concentration (mg/L) via the reference method (microdilution) and flow cytometry (FASTcolistin MIC). MICs within essential agreement (within +/−1 dilution of reference MIC) are highlighted in light grey and MIC identical in both tests are in dark grey. EUCAST and CLSI breakpoints are shown as lines. MIC ≤ 2 mg/L means Susceptible for EUCAST and Intermediate for CLSI; MIC ≥ 4 is resistant for both EUCAST and CLSI protocols.

	Reference method MIC (mg/L) (microdilution)										
FASTcolistin MIC (mg/L)		0.125	0.25	0.5	1	2	4	8	16	32	>64
	0.25	1	**2**	3							
	0.5		4	**8**	9						
	1		1	14	**10**	6					
	2				15	**17**					
	4						**5**				
	8							**1**			
	16										
	32										
	≥64										**4**

## Data Availability

Data are contained within the article.
